# Health‐related quality of life in stage III‐IV melanoma treated with targeted therapy or immunotherapy: A systematic review on the adequacy of reporting and clinical issues in phase III randomized controlled trials

**DOI:** 10.1002/cam4.5183

**Published:** 2022-08-28

**Authors:** Chen Chen, Zehua Wang, Yan‐Ru Qin

**Affiliations:** ^1^ Department of Oncology The First Affiliated Hospital of Zhengzhou University Zhengzhou China; ^2^ Cancer Institute, University College London London UK

## Abstract

Cutaneous melanoma represents around over 90% of all melanoma. With more effective treatments able to extend patients' survival, health‐related quality of life (HRQOL) is increasingly becoming an important endpoint in cancer clinical trials. They are often secondary outcomes measured in phase III randomized controlled trials and their implementation, collection, analysis, and reporting can be challenging methodologically. For these reasons, an increasing number of international recommendations introduced the standards regarding the conduct of HRQOL. In this systematic review, we appraise the adequacy of HRQOL reporting in phase III randomized controlled trials of stage III‐IV cutaneous melanoma and the clinical issues of immunotherapy and small‐molecular‐targeted therapy on HRQOL. Our search strategy totally got 55 articles, and only 13 studies met all inclusion criteria. Findings suggest that most treatments did not yield significant improvements in HRQOL but kept baseline levels, accompanied by prolonged survival and acceptable toxicity. Except for some existing limitations, reporting of HRQOL has made encouraging progress during the period covered by our search, but some aspects still need further optimization.

## Graphical abstract

In this systematic review, we appraise the adequacy of health‐related quality of life reporting in phase‐III randomized controlled trials of stage III‐IV cutaneous melanoma, and the clinical issues of immunotherapy and small‐molecular targeted therapy on health‐related quality of life. The potential effect of reporting might yield new insights into clinical decision making.

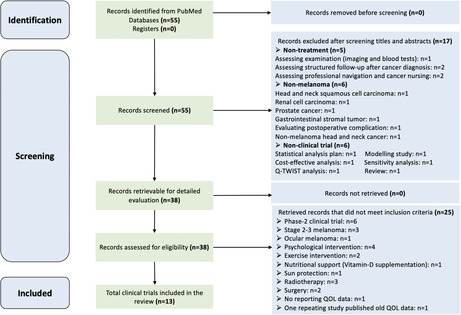

## 1 INTRODUCTION

Melanoma accounts for about 75% of skin cancer‐related deaths,^1^ of which cutaneous melanoma is the most common type representing over 90% of all cases.^2^ The incidence has been rising rapidly over the last few decades^3^ and varies in a specific geography.^4^ In 2020, melanoma is the 17th most common cancer worldwide—with an estimated 324,635 new cases and 57,043 deaths.^5^ Overexposure to ultraviolet light leading to DNA damage is an important environmental factor.^6^ The current therapy for melanoma aims to control disease symptoms and improve health‐related quality of life (HRQOL).

**FIGURE 1:**
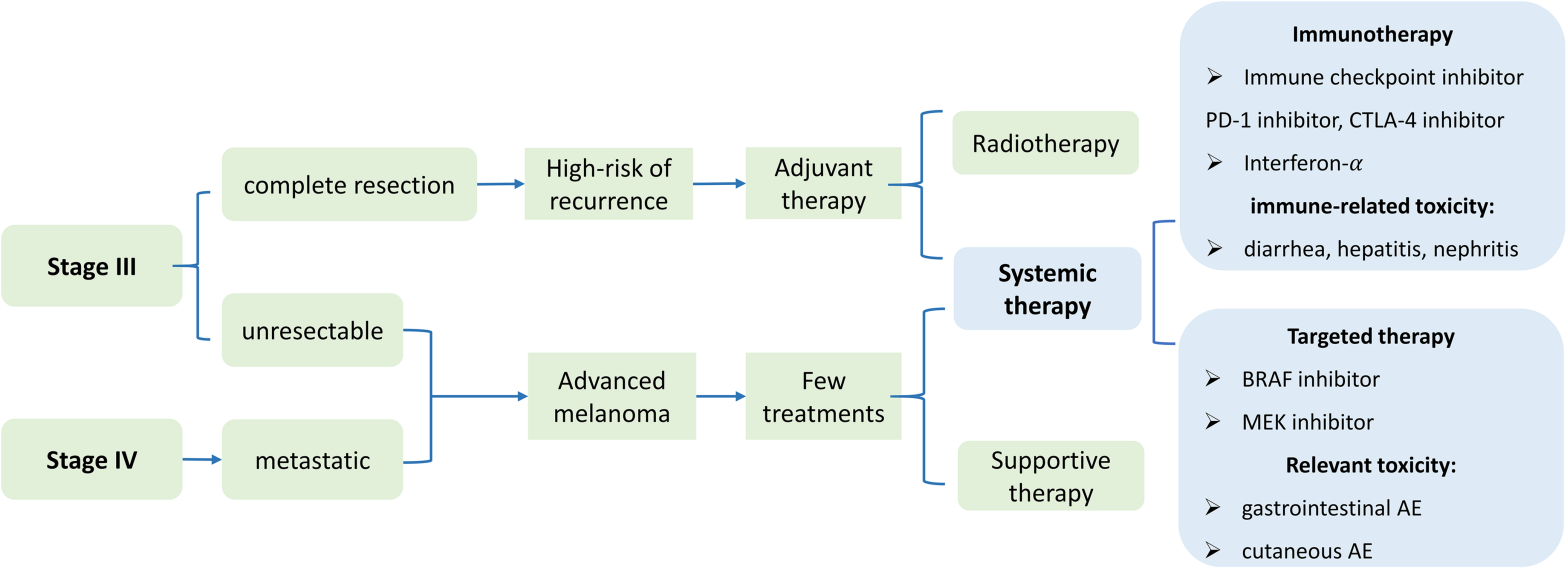
Treatment pathways for stage III‐IV cutaneous melanoma.

Cutaneous melanoma is characterized by a chronic, life‐threatening disease that could impinge on HRQOL. For clinical purposes, staging melanoma is based on the 8th edition AJCC.^7^ Most cutaneous melanoma are diagnosed with primary tumors (stage I–II) and could be successfully removed by surgical intervention with adequate margins.^8^ However, patients diagnosed with stage III‐IV melanoma tend to have poor prognoses because of increased mortality risk, high recurrence rate, and low response rate to chemotherapy. First, a recent study reported that there is an increased mortality risk with stage III–IV melanoma based on 5‐year and 10‐year survival rates,^9^ respectively, 82% and 64% for stage IIIA, 64% and 54% for stage IIIB, 46% and 34% for stage IIIC, 40% and 35% for stage IV. Second, recurrence is another challenge to long‐term prognosis. As for “resected stage III melanoma,” over 50% of patients after surgery would relapse or die at 5 years.^10^ Third, unresected stage III melanoma is generally treated similar to stage IV melanoma, both of which show low response rates to Dacarbazine—the only approved chemotherapy by the FDA.^11^ Therefore, stage III–IV melanoma has been one of the most challenging cancers to treat.^12^ The treatment pathway with advanced disease is shown in Figure 1.

Recently, the emergence of small‐molecular‐targeted therapy and immunotherapy successfully improved the overall survival^13^ but also introduced treatment‐related toxicity, which could negatively affect HRQOL. Small‐molecular‐targeted agents are specifically used to treat advanced patients with BRAF V600E/K mutations, such as vemurafenib, dabrafenib, trametinib, and cobimetinib.^14^ The most recent development was immune checkpoint inhibitors (CPI), such as ipilimumab, nivolumab, and pembrolizumab.^14^ However, these promising drugs might present a different toxicity profile from chemotherapy. Specifically, immune CPIs are associated with immune‐related adverse events (irAEs),^15^ such as colitis, nephritis, and hepatitis. Gastrointestinal and cutaneous toxicity, like photosensitivity, frequently occurs in patients undergoing small‐molecular‐targeted agents.^16^ While appraising these new drugs, it is essential to consider HRQOL data in conjunction with efficacy and safety profiles.

HRQOL assessment is essential and informative. HRQOL is considered as patient‐reported outcomes (PROs) that present independent prognostic factors to indicate the tumor burden, patients' daily living abilities, and clinical outcomes of interventions.^17^ Theoretically, HRQOL could be viewed as personal satisfaction with life, possibly affected by physical capacity, psychological status, social relationships, religious beliefs, and even cultural backgrounds. However, there are three limitations to HRQOL assessment: First, relevant implementation, data collection, analysis, and final reporting face methodological challenges without consensus. Second, HRQOL results can be affected by multiple biases.^18^ Third, many symptoms and mental issues might not be fully considered in existing HRQOL instruments (EORTC QLQ‐C30, EQ‐5D, and FACT) because most of them are designed for evaluating the health status of patients with chemotherapy, such as fatigue, nausea, vomiting, and appetite loss. As mentioned above, new therapies are associated with a different toxicity profile compared with chemotherapy. Explicitly, it is uncertain whether the occurrence of rash caused by CPI would lead to a corresponding QOL decrease on the function (emotional or social) subscale.

These challenges motivate more initiatives to optimize data collection and reporting in the context of melanoma trials. Increased awareness and interest in PRO have dramatically driven the consensus of international recommendations to improve the conceptualization of HRQOL reporting. Specifically, SPIRIT‐PRO^19^ and CONSORT‐PRO^20^ guidelines aim to support good reporting of the trial protocol and final publication, whereas SISAQOL^21^ recommendations focus on high‐quality QOL design and analysis, representing a crucial advancement toward international standards for the analysis of HRQOL endpoints in cancer randomized controlled trials.

In this systematic review, we appraise HRQOL measurements and presentations of all identified trials and potentially explore medical implications for future practice. One similar systematic review evaluating the quality of HRQOL reporting in uveal melanoma has previously been reported.^22^ However, the adequacy of HRQOL measurements of cutaneous melanoma has not been systematically reviewed. Further, a comprehensive consideration of efficacy, toxicity, and HRQOL caused by novel agents is still an unfilled research field. To our best knowledge, this is the first systematic review with the latest QOL data in clinical trials (between 2015 and 2021) to focus on stage III–IV cutaneous melanoma patients who underwent immunotherapy or targeted therapy. Consequently, we aim to investigate whether the adequacy of HRQOL reporting has made progress and whether novel treatment options bring better or noninferior HRQOL profiles.

## 2 METHODOLOGY

Our systematic review followed the PRISMA 2020 guideline,^23^ ensuring more transparent, complete, and precise reporting.

### 2.1 Eligibility criteria

Predefined inclusion criteria were phase III randomized controlled trials of patients with stage III–IV cutaneous melanoma undergoing novel systemic treatments (KRAS‐ or MEK‐targeted inhibitors, immune CPIs, interferon‐*α*, or combinations). The selected studies used HRQOL as a primary or secondary or exploratory endpoint and must be published in English Oncology journals.

Predefined exclusion criteria were any RCTs that evaluated nonantitumor treatments (psychological education, exercise intervention, sun protection, or nutritional support) and investigated traditional therapies alone (observation, chemotherapy, radiotherapy, or surgery). Any publications that were not randomized controlled trials (review, modeling study, statistical analysis, and cost‐effective analysis). Studies were ruled out if the HRQOL reports lacked or questionnaires were not completed by patients themselves.

### 2.2 Search strategy

We searched for all phase III randomized controlled trials using HRQOL as an endpoint and evaluating systemic therapies in patients with stage III–IV cutaneous melanoma, published between January 1, 2015 and May 1, 2021 regardless of their start or finishing date. The last search was completed on June 1, 2022.

We searched PubMed with the terms (“quality of life”[MeSH Terms] OR “quality of life”[All Fields] OR “qol”[All Fields] OR “health related quality of life”[All Fields] OR “hrqol”[All Fields] OR “patient reported outcome measures”[MeSH Terms] OR “patient reported”[All Fields] OR “patient reported outcomes”[All Fields] OR “pro”[All Fields]) AND (“melanoma”[MeSH Terms] OR “melanoma”[All Fields] OR “melanomas”[All Fields] OR “melanocarcinoma”[All Fields] OR “melanocarcinomas”[All Fields]) AND (“randomized”[All Fields] OR “randomized”[All Fields] OR “randomization”[All Fields] OR “randomization”[All Fields] OR “randomly”[All Fields]) AND (“2015/01/01”[Date ‐ Publication]: “2021/05/01”[Date ‐ Publication]). We limited the article search type to “Randomized controlled Trial.”

### 2.3 Selection process

Two independent reviewers (CC and ZH W) screened titles and abstracts, followed by scrutinizing full texts. And a senior researcher (YR Q) made the final decision in case of disagreement. One reviewer (CC) extracted data from identified trials and the others (ZH W and YR Q) checked.

### 2.4 Data extraction

We evaluated all identified phase III randomized controlled trials and extracted data with the same criteria comprising four information classifications:

- *key characteristics of RCT*: trial name, year of publication, authors, treatment outline, study location, sample size, cancer stage, disease condition, and endpoint.
- *A summary of HRQOL assessment tools employed*.
- *The quality of HRQOL reporting*.
- *Clinical impacts of novel agents on HRQOL*: efficacy, toxicity, and HRQOL profile.

### 2.5 Study risk of bias assessment

The Centre for Reviews and Dissemination guidance (CRD)^24^ was employed to check systematic deviations. The responses are limited to Yes/No/Partially/Not stated/Not applicable. The overall score for each study could be calculated as one “Yes” equals one point. The risk of bias was assessed by three independent reviewers (CC, ZH W, and YR Q).

### 2.6 Data syntheses

For the primary objective, the adequacy of HRQOL reporting was appraised with a checklist referred to as CONSORT‐PRO,^20^ including whether the authors explained the rationale for the choice of a specific instrument, proposed a prior hypothesis concerning HRQOL, described baseline assessment and instrument administration; whether they adequately covered all domains of HRQOL; and which manners they choose to present HRQOL data. The responses for each item include “Yes”/“No”/“Limited.” There is a total of seven assessment items. Each “Yes” is equal to one point. The total number of “Yes” adds up to the score of each study. The final score ranges from 0 to 7. We would appraise the reporting of HRQOL using this checklist.

For the secondary objective, we extracted the efficacy (progression‐free survival [PFS]/recurrence‐free survival [RFS]), toxicity, and HRQOL data from identified studies. When analyzing the magnitude of HRQOL differences between treatment modalities, both statistical significance and clinical relevance would be considered. The *p* value of less than 0.05 was traditionally taken to be the cutoff to conclude statistical significance. HRQOL changes exceeding the boundary of the prespecified threshold were defined as minimum clinically important differences (MCIDs). We would explore the associations among efficacy, toxicity, and HRQOL data sets.

## 3 RESULTS

### 3.1 Study selection

**FIGURE 2:**
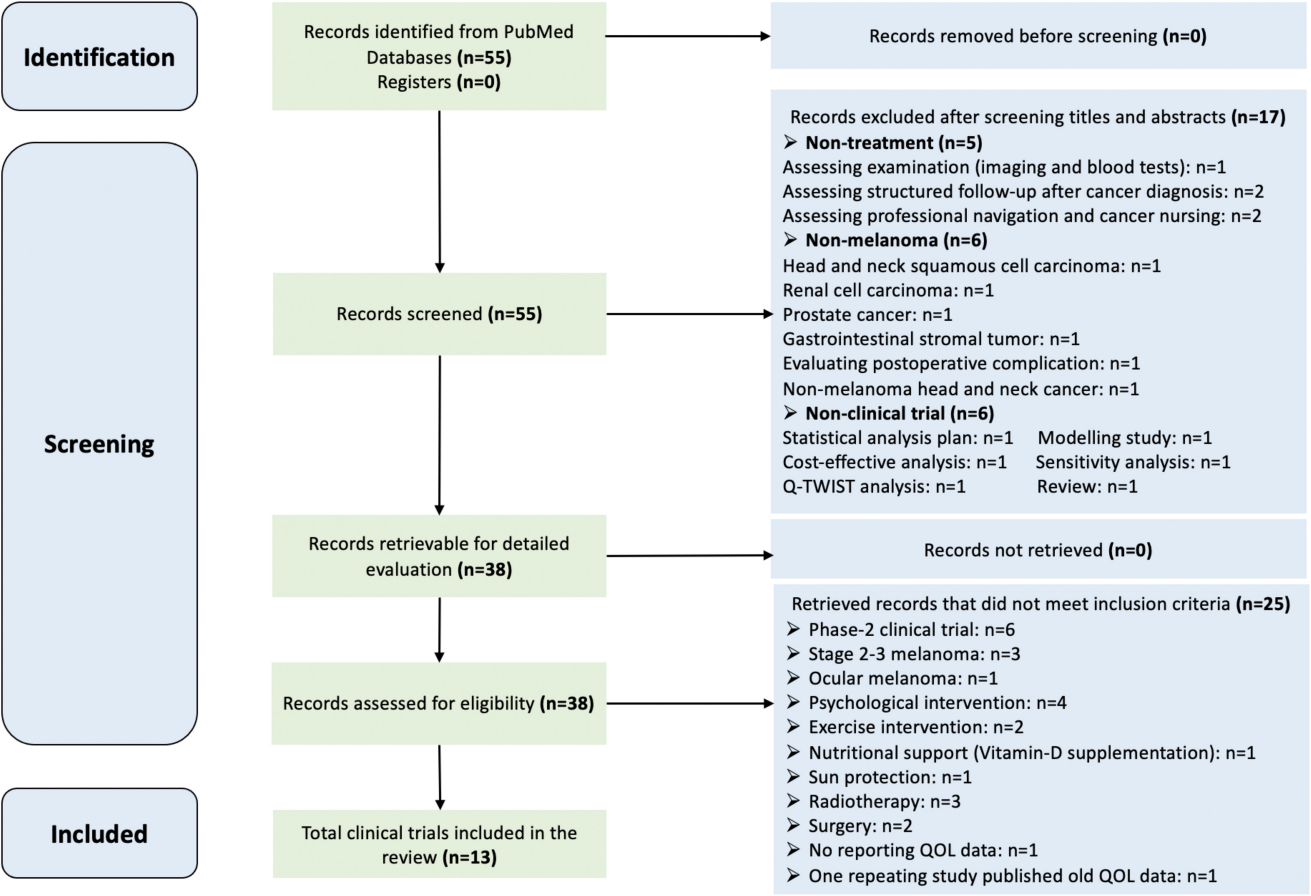
Flow diagram of the study selection.

Our search strategy totally identified 55 publications, but only 13 (23.6%) were fully eligible for this systematic review. The other 42 (76.4%) were precluded based on the criteria detailed above. The PRISMA 2020 flow diagram (Figure 2) indicated the selection process.

### 3.2 Key characteristics of identified studies

Table 1 provided an overview of all included publications.

**TABLE 1:** An overview of all included studies

Trial Name	Author/Year	Experimental treatment	Control treatment	Country	No. Patients	Cancer stage	Disease condition	Endpoint
Immune checkpoint inhibitor								
EORTC 1325‐MG	(Bottomley, 2021)^25^	Pembrolizumab	Placebo	23 countries	1019	Stage III	Complete resection High‐risk of relapse	Primary: RFS Secondary: DMFS, OS Exploratory: QOL
CheckMate 238 + EORTC 18071	(Hemstock, 2020)^26^	Nivolumab	Placebo	Multinational	906 951	Stage III–IV Stage III	Complete resection High‐risk of relapse	Primary: RFS Secondary: HRQzOL
CheckMate 067	(Larkin, 2019)^27^	Arm‐1: Nivolumab‐plus‐Ipilimumab Arm 2: Nivolumab	Ipilimumab	Australia, Europe, Israel, New Zealand, and North America	945	Stage III‐IV	Unresectable or metastatic BRAF mutation	Primary: PFS, OS; Secondary: objective response rate, descriptive efficacy; HRQOL
EORTC 18071	(Coens, 2017)^28^	Ipilimumab	Placebo	19 countries	951	Stage III	Complete resection High‐risk of relapse	Primary: RFS Secondary: HRQOL
Keynote‐006	(Petrella, 2017)^29^	Pembrolizumab	Ipilimumab	16 countries	776	Stage III‐IV	Unresectable or metastatic	Primary: PFS, OS; Exploratory: HRQOL
CheckMate 238	(Weber, 2017)^30^	Nivolumab	Ipilimumab	25 countries	906	Stage III‐IV	Complete resection High‐risk of relapse	Primary: RFS Secondary: OS, Safety, HRQOL
CheckMate 066	(Long, 2016)^31^	Nivolumab	Chemotherapy	Europe, Israel, Australia, Canada, and South America	418	Stage III‐IV	Unresectable or metastatic No BRAF mutation	Primary: OS; Secondary: PFS, objective response rate, HRQOL, PD‐L1 expression
Small‐molecular targeted therapy								
COMBI‐AD	(Drik, 2019)^32^	Dabrafenib plus Trametinib	Placebos	25 countries	870	Stage‐III	Complete resection High‐risk of relapse BRAF mutation	Primary: RFS Secondary: OS, DMFS, freedom from relapse, safety Exploratory: HRQOL
coBRIM	(Brigitte, 2018)^33^	Cobimetinib plus Vemurafenib	Placebo plus Vemurafenib	19 countries	495	Stage III‐IV	Unresectable or metastatic BRAF mutation	Primary: PFS Secondary: OS, objective response, QOL, response duration
COMBI‐V	(Grob, 2015)^34^	Dabrafenib plus Trametinib	Vemurafenib	193 centers worldwide	704	Stage III‐IV	Unresectable or metastatic BRAF mutation	Primary: OS; Secondary: PFS, safety, duration of response, overall response; Exploratory: HRQOL
COMBI‐d	(Drik, 2015)^35^	Dabrafenib plus Trametinib	Dabrafenib	Multinational	423	Stage III‐IV	Unresectable or metastatic BRAF mutation	Primary: PFS Secondary: HRQOL, OS, response rate and duration, safety, pharmacokinetics
Interferon‐α								
Sunbelt	(Egger, 2016)^36^	Interferon	Observation	Multinational	490	Stage III	Complete resection	Primary: HRQOL Exploratory: OS, RFS
DeCOG	(Peter, 2015)^37^	Intermittent high‐dose IFN‐α‐2b	Standard high‐dose IFN‐α‐2b	German, Australia, Greek, Swiss	649	Stage III	Complete resection	Primary: DMFS Secondary: RFS, OS, Safety, QOL

*Note*: Geography factor is about the countries or regions of patients involved in the trials. Melanoma staging is based on the 8th edition American Joint Committee (AJCC). Disease condition indicated whether patients received complete surgery or not; whether patients have a high risk of recurrence; whether patients have BRAF mutations. Endpoint description indicated which item was selected as a primary, secondary, or exploratory endpoint in the identified clinical trials.

Abbreviations: DMFS, distant metastasis‐free survival; HRQOL, health‐related quality of life; OS, overall survival; QOL, quality of life; PFS, progression‐free survival; RFS, recurrence‐free survival.

All 13 studies are international, multicenter, phase III RCTs involving a total of 8646 patients. The trial size ranged from 418 to 1019 patients. Of the 13 studies' interventions, 7 (53.8%) evaluated immune CPI, 4 (30.8%) evaluated small‐molecular‐targeted therapy, and 2 (15.4%) evaluated interferon. Seven (53.8%) studies focused on patients with resected, high‐risk stage III melanoma; 6 (46.2%) studies assessed patients with unresectable stage III or metastatic stage IV melanoma. HRQOL was a primary endpoint in only one trial (7.7%), a secondary endpoint in 8 (61.5%), and an exploratory endpoint in the remaining four (30.8%). All 13 articles were published in high‐impact journals.

### 3.3 Quality of evidence of all 13 RCTs

Table 2 outlined the critical appraisal of all involved studies.

**TABLE 2:** Quality of evidence of included randomized controlled trials using the appraisal tool

Trial	Randomization			Comparability		Eligibility		Blinding				Missing data			Quality assessment (max = 14)
	Truly random	Allocation concealment	Number stated	Presented	Achieved	Criteria specified	Co‐interventions	Assessors	Administration	Participants	Success	Reported	Baseline compliance >80%	Reasons stated	
(Bottomley, 2021)	Yes	Yes	Yes	Yes	Yes	Yes	Yes	NS	Yes	Yes	NS	Yes	Yes (93.60%)	Yes	12
(Hemstock, 2020)	Yes	NS	Yes	Yes	NS	Yes	Yes	NS	Yes	Yes	NS	No	NS	NS	7
(Larkin, 2019)	Yes	Yes	Yes	Yes	Yes	Yes	No	NS	Yes	Yes	NS	Yes	Yes (90.26%)	Yes	11
(Coens, 2017)	Yes	Yes	Yes	Yes	Yes	Yes	Yes	NS	Yes	Yes	NS	Yes	Yes (94.00%)	Yes	12
(Petrella, 2017)	Yes	Yes	Yes	Yes	Yes	Yes	NS	No	No	No	No	Yes	Yes (98.84%)	Yes	9
(Weber, 2017)	Yes	Yes	Yes	Yes	Yes	Yes	Yes	NS	Yes	Yes	NS	No	NS	NS	9
(Long, 2016)	Yes	Yes	Yes	Yes	Yes	Yes	No	NS	Yes	Yes	NS	Yes	Yes (67.46%)	NS	10
(Drik, 2019)	Yes	Yes	Yes	Yes	Yes	Yes	Yes	NS	Yes	Yes	NS	Yes	Yes (98.00%)	NS	11
(Brigitte, 2018)	Yes	Yes	Yes	Yes	Yes	Yes	NS	NS	Yes	Yes	NS	Yes	Yes (96.90%)	NS	10
(Grob, 2015)	Yes	Yes	Yes	Yes	Yes	Yes	NS	No	No	No	No	Yes	Yes (97.00%)	NS	8
(Drik, 2015)	Yes	Yes	Yes	Yes	Yes	Yes	NS	NS	Yes	Yes	NS	No	Yes (95.98%)	NS	9
(Egger, 2016)	Yes	NS	Yes	NS	NS	Yes	Yes	NS	NS	NS	NS	Yes	Yes (100%)	NA	6
(Peter, 2015)	Yes	NS	Yes	Yes	Yes	Yes	Yes	NS	NS	NS	NS	No	NS	NS	6

*Note*: Yes, item adequately addressed; no, item not adequately addressed; NS, not stated; NA, not applicable. The number of “yes” in each row is counted as the score of the quality assessment of each study, assessing the risk of bias; the score ranges from 0 to 14.

All studies reported sufficient randomization information and clearly stated the number of participants in each arm. Ten of 13 studies (77%) reported adequate concealment of allocation. Twelve (92%) presented baseline comparability of participants. Eleven (85%) reported the balance of baseline characteristics between arms. Nine (69%) were double‐blinded trials, and the remaining four trials (31%) were reported as “open.” Blinging of assessors was not stated in all 13 studies. Ten (77%) reported baseline compliance of over 80% of participants. Nine studies (69%) reported missing data, but only 4 (31%) stated reasons.

The quality of evidence varied based on CRD (2009).^24^ The overall score of eligible studies ranged from 6 to 12 out of a maximum of 14. Six studies got more than 10 points. The quality of evidence is robust enough to support our systematic review.

### 3.4 The adequacy of health‐related quality‐of‐life reporting

Table 3 summarized all HRQOL instruments employed.

**TABLE 3:** Assessment tools employed by 13 melanoma trials in this systematic review

Instrument	Type	Culturally validated	Number of studies	Number of patients
EORTC QLQ‐C30	Cancer specific	Yes	10 (71.4%)	7286
EQ‐5D‐3L	Generic	Yes	7 (50%)	4619
FACT‐M	Melanoma specific	Yes	1 (7.1%)	704
PC and QOL questionnaire	Self‐defined	No	1 (7.1%)	490

*Note*: The type of instrument indicates the scope of application of the selected questionnaire. Number of studies shows how many identified studies used these instruments, respectively. Number of patients shows the total sample sizes that are involved in each QOL instrument.

HRQOL measurement was conducted by a similar methodology but with diverse QOL instruments. Of all publications we determined, in order of the frequency of QOL questionnaire used, they were respectively EORTC QLQ‐C30, EQ‐5D, FACT‐M, and a self‐defined tool. Particularly, 5 of 13 studies (38.5%) employed more than one HRQOL instrument. Of these 5 trials selected both EORTC QLQ‐C30 and EQ‐5D questionnaires, of which one study additionally employed a melanoma‐specific (FACT‐M) instrument. Altogether, 12 of 13 studies (92%) used culturally validated instruments to measure melanoma patients' HRQOL.

Table 4 evaluated the adequacy of HRQOL reporting in 13 studies. We defined validity and reliability as to whether QOL instruments have robust psychometric properties and could be applicable in melanoma patients, which is essential for participants from different cultures attending clinical trials for new drug approval. For 11 reports (84.6%), the validity and reliability of the tools were referenced with corresponding validation studies. Of the remaining 2 (15.4%), there was no information regarding the validity and reliability, although one study^26^ selected an internationally validated questionnaire—EORTC QLQ‐C30. However, the other one^36^ used an unvalidated tool—physical condition and QOL questionnaires that were originally developed, which seemed to be less robust to reflect patient‐reported health status.

**TABLE 4:** Assessment methods for health‐related quality of life.

Author/Year	HRQOL instruments	Validity and reliability referenced	Rationale for tools	Hypothesis stated	Baseline QOL assessment	Timing of assessment	Instrument administration	Adequacy of scales covered	Quality of QOL measures	Presentation ways
Immune checkpoint inhibitor										
(Bottomley, 2021)	EORTC QLQ‐C30	Yes	Yes	Yes	Yes	Yes	Yes	Yes	7	Table; Figure; Text
(Hemstock, 2020)	EORTC QLQ‐C30	No	No	No	No	No	No	Yes	1	Figure; Text
(Larkin, 2019)	EORTC QLQ‐C30 EQ‐5D	Yes	Yes	No	Yes	Yes	No	Limited	4	Table; Figure; Text
(Coens, 2017)	EORTC QLQ‐C30	Yes	Yes	Yes	Yes	Yes	Yes	Limited	6	Table; Figure; Text
(Petrella, 2017)	EORTC QLQ‐C30 EQ‐5D	Yes	Yes	Yes	Yes	Yes	Yes	Yes	7	Table; Figure; Text
(Weber, 2017)	EORTC QLQ‐C30 EQ‐5D	Yes	Yes	No	No	Yes	No	Limited	3	Figure; Text
(Long, 2016)	EORTC QLQ‐C30 EQ‐5D	Yes	Yes	No	Yes	Yes	No	Yes	5	Table; Figure; Text
Targeted therapy										
(Drik, 2019)	EQ‐5D‐3L	Yes	Yes	No	Yes	Yes	No	Limited	4	Table; Figure; Text
(Brigitte, 2018)	EORTC QLQ‐C30	Yes	Yes	No	Yes	Yes	Yes	Yes	6	Figure, Text
(Grob, 2015)	EORTC QLQ‐C30 EQ‐5D FACT‐M	Yes	Yes	No	Yes	Yes	No	Yes	5	Table, Figure, Text
(Drik, 2015)	EORTC QLQ‐C30	Yes	Yes	No	Yes	Yes	No	Yes	5	Table; Figure; text
Interferon‐α										
(Egger, 2016)	PC and QOL questionnaire	No	Yes	No	Yes	Yes	No	Limited	2	Table; Figure; Text
(Peter, 2015)	EORTC QLQ‐C30	Yes	No	No	No	Yes	No	Yes	3	Figure; text

*Note*: Presentation ways were assessed if the authors used figures, tables, or texts to present HRQOL outcomes. We assessed the validity and reliability of the instrument and whether the authors provided relevant data that can be retrieved from a reference. Rationale for instrument choice was assessed whether the authors explained or justified the selection process. Hypothesis statement was assessed whether the authors put forward a clear prior hypothesis to estimate potential QOL changes. Baseline assessment was assessed whether the authors reported the percentage of patients who received questionnaires at baseline. Instrument administration was assessed if the authors specified who administered the HRQOL instrument or in which clinical setting the instrument was administered. [The number of “Yes” in each row is counted as the score of the quality of QOL reporting in each study; the score ranges from 0 to 7].

The rationale for instrument selection was evaluated whether the authors justified the choice process. We defined it as introducing some basic characteristics of an instrument or applicative reasons. Eleven (84.6%) studies provided relevant details. The HRQOL domains covered by selected questionnaires were adequate enough, including both symptom and function subscales. Nevertheless, only 23.1% put forward a pretrial research hypothesis for the assumed differences in HRQOL between arms. One study—the DECOG trial,^37^ gave a hypothesis about efficacy but not relevant to QOL. Two studies—COMBI‐V^34^ and COMBI‐D^35^—explained the major reason for no hypotheses regarding QOL. The data lacked enough statistical power so the results should be considered exploratory and verified in future research.

The process, describing how the HRQOL was assessed, was generally reported with few details. But some explicit descriptions were noted. For example, 12 studies (92.3%) described the timing of assessment; 77% of studies reported baseline HRQOL assessment, and all of them demonstrated favorable compliance. The instrument administration was assessed if the authors specified who managed the QOL questionnaire or in which clinical setting the HRQOL instrument was administered. Only 4 (30.8%) reported this process in the methodology.

HRQOL measurement was adequate in 8 (61.5%) studies, reporting all scales consistent with the intended analysis described in the introduction or methodology. Conversely, we defined it to be limited if the publication did not provide detailed QOL score change of each domain or did not interpret the meaning or implication of data. Some trials focused on the global health quality‐of‐life (GHQ) or symptoms without reporting any functions. For instance, one research^36^ introduced a new instrument that only reflected the general health status without detailed scores of any subscale.

All studies reported HRQOL data in figures and texts, of which 9 reports offered tables. EORTC 1325‐MG^25^ reported HRQOL data in three manners, whereas CheckMate 238^30^ only provided texts and figures. The former tends to show more details than the latter one.

Overall, the number of Yes in each study is counted as the overall score, ranging from 0 to 7 points. Consequently, 9 studies (69.2%) scored at least 4 points. HRQOL data were accurately measured in most of the identified RCTs.

### 3.5 Clinical impacts of novel agents on HRQOL

Table 5 presented efficacy, toxicity, and HRQOL comparisons between different treatments.

**TABLE 5:** Summary of HRQOL, efficacy, and safety outcomes between different treatments.

Author/Year	Experimental treatment	Control treatment	Differences in PFS/RFS	Differences in toxicity	Differences in HRQOL			
					QOL comparisons	Statistical difference	Clinical difference	Main HRQOL outcomes
Immune checkpoint inhibitor								
(Bottomley, 2021)	Pembrolizumab	Placebo	Hazard ratio for RFS favoring pembrolizumab: 0.57^a^ [98% CI: 0.43–0.74; *p* < 0.0001] 1‐year rate of RFS: pembrolizumab: 75.4%^a^ placebo: 61.0%	Grade 3–5 AEs: pembrolizumab: 14.7% placebo: 3.4%	Mean score difference between arms	*p* > 0.05	No	No significant differences between treatment groups
(Hemstock, 2020)	Nivolumab	Placebo	Hazard ratio for RFS favoring nivolumab: 0.53 [95% CI: 0.41–0.68]^a^	Hazard ratio for any AEs favoring placebo: 1.48 [95% CI: 1.22–1.80]	Hazard ratio for the time of QOL deterioration	HR close to 1	NR	Results of the HRQOL comparisons show broadly similar results between nivolumab and placebo; 14 of the 15 comparisons were not statistically significant; Dyspnoea statistically favored placebo over nivolumab
(Larkin, 2019)	Arm 1: Nivolumab‐ipilimumab Arm 2: Nivolumab	Ipilimumab	Median PFS: combination: 11.5 months^a^ [95% CI: 8.7–19.3] nivolumab: 6.9 months^a^ [95% CI: 5.1–10.2] ipilimumab: 2.9 months [95% CI: 2.8–3.2]	AEs: combination: 96% nivolumab: 86% ipilimumab: 86%	Mean score difference between arms	*p* > 0.05	No	Nivolumab and ipilimumab combination and nivolumab alone both maintained HRQOL, and no clinically meaningful deterioration was observed over time compared with ipilimumab. During the follow‐up for survival, clinical deterioration occurred more frequently in the ipilimumab monotherapy group than in the other treatment groups.
(Coens, 2017)	Ipilimumab	Placebo	Median RFS: ipilimumab: 26.1 months^a^ [95% CI: 19.3–39.3] placebo: 17.1 months [95% CI: 13.4–21.6] Hazard ratio for RFS favoring ipilimumab: 0.75^a^ [95% CI: 0.64–0.90; *p* < 0.0013]	Immune‐related AEs in ipilimumab vs. placebo: Gastrointestinal: 16% vs. <1% Hepatic: 11% vs. <1% Endocrine: 8% vs. 0%	Mean score difference between arms	***p* < 0.05**	No	For the GHQ score, despite statistical significance, there was no clinical relevance between the two groups at any time. For other scales, worse scores were reported in the ipilimumab group compared with the placebo group; clinically relevant deterioration for some symptoms was observed at week 10, but after induction, no clinically relevant differences remained.
(Petrella, 2017)	Pembrolizumab	Ipilimumab	PFS rate: Pem every 2 weeks: 47.3%^a^ Pem every 3 weeks: 46.4%^a^ Ipilimumab: 26.5% Hazard ratio for PFS favoring both pembrolizumab regimens: 0.58 (*p* < 0.001)^a^	Grade 3–5 AEs^c^: Pem every 2 weeks: 13.3% Pem every 3 weeks: 10.1% Ipilimumab: 19.9%	Mean score difference between arms	***p* < 0.05**	No	GHQ score was better maintained with pembrolizumab than with ipilimumab. Other function and symptom scales showed similar trends.
(Weber, 2017)	Nivolumab	Ipilimumab	12‐month rate of RFS: nivolumab: 70.5%^a^ [95% CI: 66.1–74.5] ipilimumab: 60.8% [95% CI: 56.0–65.2] Hazard ratio for RFS favoring nivolumab: 0.65^a^ [95% CI: 0.51–0.83; *p* < 0.001]	Grade 3–4 AEs^c^: nivolumab: 14.4% ipilimumab: 45.9% Discontinuations due to AEs^c^: nivolumab: 9.7% ipilimumab: 42.6%	NC	NC	NC	QOL scores in the two groups remained close to baseline value without any clinically meaningful changes. No comparisons between treatment groups.
(Long, 2016)	Nivolumab	Chemotherapy	Median PFS: nivolumab: 5.1 months^a^ dacarbazine: 2.2 months Hazard ratio for PFS favoring nivolumab: 0.43^a^ [95% CI: 0.34–0.56; *p* < 0.001]	Grade 3–4 AEs^c^: nivolumab: 11.7% chemotherapy: 17.6%	Mean score difference between arms	*p* > 0.05	No	No clinical differences between treatment groups. Nivolumab maintains baseline HRQOL levels to provide long‐term survival benefits compared with chemotherapy.
Small‐molecular‐targeted therapy								
(Drik, 2019)	Dabrafenib plus Trametinib	Placebo	3‐year rate of RFS: combination: 58%^a^ placebo: 39% Hazard ratio for RFS favoring combination: 0.47^a^ [95% CI: 0.39–0.58; *p* < 0.001]	Combination vs. Placebo: At least one AE: 97% vs. 88% Serious AEs: 36% vs. 10% A new primary melanoma: 3% vs. 2%	Mean score difference between arms	*p* > 0.05	No	During treatment and long‐term follow‐up, VAS and utility scores were similar between two groups and did not differ from baseline scores. At recurrence, both VAS and utility scores significantly decreased for both groups compared to baseline scores. Dabrafenib‐plus‐trametinib did not affect patient‐reported outcome scores during or after adjuvant treatment. Preventing or delaying relapse with adjuvant therapy could be beneficial in this setting
(Brigitte, 2018)	Cobimetinib plus Vemurafenib	Placebo plus Vemurafenib	Median PFS: C + V: 12.3 months^a^ P + V: 7.2 months Hazard ratio for PFS favoring combination: 0.58^a^ [95% CI: 0.46–0.72; *p* < 0.0001]	Serious AEs: combination: 37% vemurafenib: 28%	Mean score difference between arms	*p* > 0.05	No	There were few clinically meaningful differences between the C + V and P + V across all functional domains and GHQ. Among the symptom scales, there was a clinically meaningful improvement in pain in the C + V; the only item favoring the P + V was diarrhea.
(Grob, 2015)	Dabrafenib plus Trametinib	Vemurafenib	Median PFS: combination: 11.4 months^a^ vemurafenib: 7.3 months Hazard ratio for PFS favoring vemurafenib: 0.56^a^ [95% CI: 0.46–0.69; *p* < 0.001]	Severe AEs and study‐drug discontinuations were similar in two groups. Cutaneous squamous‐cell carcinoma/keratoacanthoma^c^: 1% vs. 18%	Mean score difference between arms	***p* < 0.001**	Yes^d^	Differences in mean scores between treatment groups were significant and clinically meaningful in favor of the combination compared with vemurafenib monotherapy for most domains across all three questionnaires during study treatment and at disease progression. QOL score difference favoring combination therapy (combination‐minus‐vemurafenib): GHQ: 7.92 points EQ‐5D VAS: 7.96 points FACT‐M: 3.62 points
(Drik, 2015)	Dabrafenib plus Trametinib	Dabrafenib	3‐year PFS rate: combination: 22%^a^ monotherapy: 12% Median PFS: combination: 9.3 months^a^ monotherapy: 8.8 months Hazard ratio for PFS favoring dabrafenib: 0.75^a^ [95%: 0.57–0.99; *p* = 0.035]	Combination vs. Dabrafenib Cutaneous squamous‐cell carcinoma^c^: 2% vs. 9% Pyrexia: 51% vs. 28% Grade‐3 AEs: 6% vs. 2%	Mean score difference between arms	***p* < 0.05**	No	The combination therapy provides better preservation of HRQOL and pain improvements versus dabrafenib monotherapy, while also delaying progression. The majority of functional dimension scores trended in favor of the combination. Apart from pain scores, other symptom scores trended in favor of dabrafenib monotherapy.
Interferon‐α								
(Egger, 2016)	Interferon	Observation	Hazard ratio for DFS favoring interferon: 0.82^a^ [95% CI: 0.50–1.36; *p* = 0.45]	The toxicities from IFN therapy were substantial.	Hazard ratio for a delay in return to baseline QOL and PC scores	**HR >1** ***p* < 0.05**	NR	There were statistically differences in time to return to baseline QOL and PC scores between IFN therapy group and resection alone group. IFN therapy is associated with worse QOL outcomes compared with observation.
(Peter, 2015)	Intermittent IFN (iHDI)	Standard IFN (HDI)	Hazard ratio for RFS favoring standard IFN: 1.27 [*p* = 0.03]^b^	Early termination of treatment due to AEs^c^ iHDI: 14.8% HDI: 26.0% [*p* < 0.001]	Mean score difference between arms	***p* < 0.001**	Yes^d^	The QOL profiles for the intermittent regimen were favorable. The overall impairment on QOL was substantially less with iHDI than with conventional HDI. Score difference favoring iHDI: Fatigue at week 16: >10‐point Role function at week 16: >10‐point Physical function at week 16: 5–10 point

Abbreviations: 95% CI, 95% confidence interval; AEs, adverse events; C + V, Cobimetinib‐plus‐vemurafenib; GHQ, global health quality of life; HR, Hazard ratio; HRQOL, health‐related quality of life; MCIDs, minimum clinically important differences; NC, no comparisons between groups; NR, no data reported; OS, overall survival; P + V, Placebo‐plus‐vemurafenib; Pem, Pembrolizumab; PFS, progression‐free survival; QOL, quality of life; RFS, recurrence‐free survival.

^a^ Efficacy outcomes favoring experimental treatment.

^b^ Efficacy profile favoring control treatment.

^c^ Safety profile favoring experimental treatment.

^d^ QOL profile favoring experimental treatment with both statistical and clinical differences. QOL results in bold represent statistical differences or clinical differences.

Novel interventions responsible for prolonged survival (OS, PFS, and RFS) are also associated with treatment‐related toxicity, and a longer survival undoubtedly leads to persistent exposure to antitumor agents, all of which might influence HRQOL.

#### 3.5.1 Associations between efficacy and HRQOL

It is vital to explore the potential associations between efficacy and HRQOL. Overall, all studies reported PFS and RFS outcomes to provide evidence of efficacy, of which 9 (69%) of 13 studies used either PFS or RFS as a primary endpoint. Findings from 12 studies (92%) showed significant prolonged PFS or RFS favoring experimental interventions over the systemic use of control drugs, of which 10 studies also presented noninferior HRQOL. Thereby, most novel agents not only successfully delay the tumor progression or recurrence but also provide more benefits or no deterioration of HRQOL.

Nevertheless, a contradictory trend of efficacy and HRQOL was observed in 3 trials, respectively, named Sunbelt trial,^36^ EORTC 18071,^28^ and the DECOG trial.^37^ As a longer PFS/RFS indicates stable tumor control and fewer disease‐related symptoms, these conflicting results might challenge whether delayed tumor growth could result in better HRQOL. Notably, the Sunbelt trial^36^ demonstrated IFN therapy was associated with a few improvements in survival, resulting in poor health status and substantial toxicity compared with0 observation alone after surgery. It might be inferred that adjuvant IFN offered limited benefits for advanced melanoma, which exactly corresponded with previous research indicating IFN is unsuitable for wide use in clinical settings.

#### 3.5.2 Associations between toxicity and HRQOL

Treatment‐related toxicity served as a bias that usually complicates our judgments of HRQOL. Eight of 13 studies (62%) demonstrated more AEs occurred in the experimental groups. Only 5 cases (38%) showed a lower frequency of toxicity in favor of experimental arms, which is parallel to the noninferior HRQOL results. If only considering both improvements of HRQOL and safety, they were, respectively, derived from pembrolizumab over ipilimumab,^29^ dabrafenib‐plus‐trametinib over vemurafenib,^34^ and intermittent IFN over standard IFN.^37^ However, not all experimental drugs showed consistent safety and HRQOL outcomes. Some novel agents resulted in worse AEs but introduced stable HRQOL. This finding might challenge the direct correlation between safety and HRQOL.

#### 3.5.3 Comparisons of HRQOL between treatment groups

For 12 of 13 (92.3%) RCTs identified, statistical tests for between treatment differences in HRQOL were stated. Findings from 6 (46%) showed no statistical significance due to the big *p* value (*p* > 0.05) or no‐effect value of hazard ratio (HR = 1). Consequently, there was less evidence to support one treatment modality over another yielding significantly improved HRQOL. For the other six trials presenting statistical differences (*p* < 0.05), only 2 (15.4%) achieved the clinical meaningfulness of differences. The trials were COMBI‐V and DECOG, both in favor of novel interventions with better health status. Taken together, more HRQOL benefits of combination therapy of BRAF/MEK inhibitors were found over monotherapy; and patients in either pembrolizumab or nivolumab arm had better HRQOL maintenance versus the ipilimumab group.

In summary, most novel systemic therapies could maintain baseline HRQOL levels to provide long‐term survival benefits and manageable toxicity.

## 4 DISCUSSION

This systematic review aims to evaluate the adequacy of HRQOL reporting in phase III melanoma RCTs and the clinical effects of immunotherapy and small‐molecular‐targeted therapy on HRQOL.

### 4.1 Quality appraisal of HRQOL reporting

Our review highlights that HRQOL was comprehensively, reliably, and precisely reported in a most international phase III melanoma RCTs between 2015 and 2021, even if we also observe problematic reporting in several critical criteria.

Over half of the reports employed generic instruments—EORTC QLQ‐C30 or EQ‐5D, but only one study chose a melanoma‐specific tool—FACT‐M. Although generic tools indeed provide applicable overall QOL assessments, allowing for comparability between melanoma patients and the general population, it might be less sensitive to detect specific symptoms or functions associated with melanoma, such as sun avoidance affecting social functioning. Compared to patients with other malignancies, such as gastrointestinal tumors or lung cancer, melanoma patients are likely to undergo fewer restrictions on their physical functioning but be much more influenced mentally.^38^ The content of the FACT‐M questionnaire is especially applicable for QOL issues associated with melanoma. Another EORTC‐modified melanoma‐specific module—EORTC‐MEL38, was developed but not validated.^39^

Recognizing the discrepancies between generic and specific questionnaires, Luckett et al.^40^ stated an algorithm for selecting an appropriate one between them, depending upon which aspects of HRQOL (symptoms, functions, or emotional status) a given trial concentrates on. Collectively, the choice of a suitable instrument needs a tradeoff among the sample population, research objectives, and psychological properties of the questionnaire.^38^

Our review highlights that 84.6% of studies stated the rationale for employing a particular questionnaire. As the choice of the questionnaire affects the data collection, analysis, reporting, and interpretation, it requires accurate justification to test its availability. This demand might be more pronounced when applying melanoma‐specific modules, like FACT‐M and EORTC QLQ‐MEL 38. Since such supplementary modules bring extra psychological burdens to patients, their application needs another round of validation before practice.

Frustratingly, the research hypotheses that refer to the HRQOL were rarely proposed (only 23.1%). The design of the HRQOL endpoint depends upon the research questions to be answered. Although the primary endpoint usually drives the sample size and stratification to conduct analyses, other study characteristics (selection of instruments, method of administration, and data collection) should be tailored to a pretrial hypothesis. Moreover, an upfront hypothesis deserves a clear illustration to develop a statistical plan for each trial. Nonpredefined hypotheses are likely to result in misleading QOL reports, such as overemphasizing statistical significance than clinical relevance; or complicating our evaluation to judge whether the scales they selected were appropriate and justified. An initial research hypothesis is becoming an indispensable need for a good trial design, which determines the generalizability and credibility of findings.^41^

The inclusion of baseline assessment should be mandatory in all clinical trials. Its absence could result in worse compliance, and therefore imperil the validity of findings. Most studies (92.3%) reported the timing of assessment, but few (only 30.8%) described instrument administration. If investigators did not report who and how to manage assessment tools, we were uncertain whether all studies were administrated reliably and whether all data were comparable. All trials should be required to inform instrument administration and rigorously comply with this strategy.

The idea of checking whether texts, graphs, or tables are reported is to examine whether data are presented in a straightforward, clear, and complete way or not. The study with tables and graphs presented more details compared with the one that only provided texts. Explicitly, tables tend to offer summarized information, while graphs are often used to demonstrate dynamic changes of QOL in each domain.

Selective reporting bias should be emphasized. A research^42^ revealed patients might not report pain if they considered that it did not originate from melanoma. This phenomenon might be more distinct in unblinded trials. Researchers should cautiously analyze comparisons between open‐label and double‐blinding studies. Moreover, in comparison with Germans, Norwegian adults reported a slightly higher GHQ score of EORTC QLQ‐C30^43^ but substantially more symptoms of constipation and diarrhea.^44^ Geography may be another obstacle affecting the reliability of findings.

Some designs of the HRQOL measurement are flawed. Due to sun avoidance behaviors, the changing lifestyles, fear of relapse, and increased psychosocial burdens are likely to constitute important domains of HRQOL analysis that are generally not fully measured in any existing instrument.

The heterogeneity of these trials restricted cross‐trial comparisons from an HRQOL perspective. These inconsistencies may be attributed to the absence of standardized questionnaires and statistical analysis. The facets of HRQOL assessed the marking system applied, and the outcomes reported undoubtedly vary in specific ways. Not all outcomes were comparable since not all studies were presented in the same way. Despite cross‐trial comparisons bringing interesting clinical information, the interpretation of HRQOL outcomes is challenging and should be done carefully.

Evidently, HRQOL can provide information about the experience of melanoma patients in the trial, such as symptom control and function improvement caused by systemic therapy. Immunotherapy and small‐molecular‐targeted therapy generally bring better efficacy and different toxicity profiles in contrast to chemotherapy. These self‐reported HRQOL data could offer valuable information regarding the effect of novel agents on advanced melanoma patients. Medical consensus depends on publications to provide a bunch of evidence that will support the future clinical decisions. Consequently, inadequate or inaccurate HRQOL reporting would seriously affect the reliability and credibility of these outcomes, which can hinder the use of HRQOL data for clinical decision‐making.

### 4.2 Clinical issues of new drugs on HRQOL

Our findings also manifest that, so far, there was less evidence to support one treatment option over another producing significantly improved HRQOL scores. A large percentage of studies showed no clinical differences between arms, and most novel therapies could maintain baseline HRQOL levels to provide long‐term survival and manageable toxicity. Although absolute recommendations on medical practice are hard to establish from our current review, it is likely to infer that anti‐PD‐1 inhibitors and a combination of BRAF/MEK inhibitors are hopeful candidates for wide use in melanoma treatment.

These novel treatment modalities tend to provide potent efficacy. However, our review might not support the positive correlation between efficacy and HRQOL. On the one hand, the sample size is too small to support regression analysis. On the other hand, we observed inconsistent outcomes of these two endpoints reported in 3 studies. That is to say, not all systemic drugs with potent efficacy also showed pleasant HRQOL. Meanwhile, as few studies focused on the relationship between PFS and HRQOL, PFS serving as a potential surrogate for HRQOL remains controversial and uncertain.^45^ There are unmet needs to determine the association between efficacy and HRQOL. Specific regression analyses are needed in future study.

As health status is frequently impaired by AEs, especially for immune‐related AEs, the association between toxicity and HRQOL is complicated. Less toxicity could lead to better health status, but it does not mean more AEs would necessarily cause a decrease in HRQOL score. Although HRQOL deterioration occurred less frequently in a nivolumab‐plus‐ipilimumab group than the ipilimumab group, a higher rate of AE was observed in the combination arm over time. It seems to contradict the idea that toxicity negatively affects HRQOL.^18^ Thereby, novel agents might lead to more AEs, but not necessarily a poorer HRQOL. Also, a longer follow‐up HRQOL assessment^33^ showed the incidence of toxicity decreased with sustained therapy, as the early onset of these AEs could be resolved by clinical management.^46^ Noteworthily, in most studies, patients undergoing higher‐grade AEs were not required to finish the HRQOL questionnaire before the withdrawal. By doing so, PRO data would not adequately reflect patients' AEs.^33^ To address these concerns, researchers need to involve all participants who discontinued treatment owing to AEs in the final HRQOL analysis, which would be essential steps for reporting precise data and investigating possible correlations.

### 4.3 Limitations of the review process

Our systematic review has some limitations. We only focused on RCTs. Instead, we did not review protocols, statistical analysis plans, or other types of research. It restricted us to comment on the entire study. We only assessed articles published in English. However, as important studies are generally reported in English journals, our review could cover the most meaningful findings. Another limitation is some subjective criteria were employed to judge the quality of HRQOL reporting. But each criterion was appraised by two independent reviewers under the supervision of a senior researcher. We believe our review is fairly consistent. Furthermore, we might mistakenly exclude some studies that only measured HRQOL subscales or that did not mention the keyword of HRQOL and its abbreviations. We only evaluated HRQOLs that have been published. There is no indication of the number of unreported studies and no explanations for the absence, such as journal‐driven or researcher‐driven limitations.

### 4.4 Implications from this review

Applying HRQOL data to guide precision medicine, the precise, adequate, and unified approaches to report HRQOL are fairly important, which need strict conduction with standardized methodology, thoughtful study design, active participation of patients from beginning to end, high‐quality data collection, and transparent trial reporting. With more advances in reporting HRQOL, the inclusion of HRQOL in large clinical trials of advanced melanoma will contribute to clinical decision‐making.

## 5 CONCLUSION

Given the high cost and complexity of international melanoma RCTS, and the substantial demand for safe therapeutic options, healthcare decisions should be made cautiously by physicians, patients and their families, abiding by reliable, validated, scientific recommendations for collection, analysis and reporting. The medical consensus relies on a bunch of publications to assemble evidence that contributes to guiding future clinical decisions. Many collaborative groups have boosted standardization. In brief, the adequacy of HRQOL reporting in Phase III RCT has made significant progress, despite some aspects needing further optimization. Also, most immune CPIs and small‐molecular‐targeted agents showed robust efficacy, tolerable toxicity, and preserved HRQOL.

## AUTHORS' CONTRIBUTION

YQ designed the study and reviewed the manuscript. CC and ZW participated in the study design and wrote the original draft of the manuscript. CC was mainly responsible for the design of tables and figures. ZW was involved in document retrieval and review. All authors agreed to the submission of the final manuscript.

## FUNDING INFORMATION

This work was funded by the National Natural Science Foundation of China (grant no. 81872264).

## CONFLICTS OF INTEREST

The authors declare that they have no conflict of interest.

## ETHICS STATEMENT

The ethical approval is not applicable in my systematic review.

## Data Availability

Data sharing is not applicable to this article as no new data were created or analyzed in this study.
